
JOURNAL INFORMATION
==============================
NLM Title Abbreviation: Biospektrum (Heidelb)
Journal ID: Biospektrum (Heidelb)
NLM Journal ID: 9615685

Biospektrum

ISSN: 0947-0867
EISSN: 1868-6249

ARTICLE INFORMATION
==============================
PMCID: PMC7318723
PMID: 32834539
DOI: 10.1007/s12268-020-1401-7
Article ID: 1401
Article version: 1
Subjects: Anwendungen & Produkte

Multiklonale Antikörper als Ersatz für Zweitantikörper aus Seren

Wenzel Esther Veronika 1
Russo Giulio 1
Dübel Stefan s.duebel@tu-bs.de hello@abcalis.com https://abcalis.com
1 2
1 grid.6738.a 0000 0001 1090 0254 Institut für Biochemie, Biotechnologie und Bioinformatik, Technische Universität Braunschweig, Spielmannstraße 7, D-38106 Braunschweig, Deutschland
2 Abcalis GmbH, Inhoffenstraße 7, D-38124 Braunschweig, Deutschland
Electronic publication date: 2020 Jun 26
Print publication date: 2020
Volume: 26
Issue: 4
First page: 416
Last page: 417
Copyright: © Die Autoren 2020
License: Open Access: Dieser Artikel wird unter der Creative Commons Namensnennung 4.0 International Lizenz veröffentlicht, welche die Nutzung, Vervielfältigung, Bearbeitung, Verbreitung und Wiedergabe in jeglichem Medium und Format erlaubt, sofern Sie den/die ursprünglichen Autor(en) und die Quelle ordnungsgemäß nennen, einen Link zur Creative Commons Lizenz beifügen und angeben, ob Änderungen vorgenommen wurden. Die in diesem Artikel enthaltenen Bilder und sonstiges Drittmaterial unterliegen ebenfalls der genannten Creative Commons Lizenz, sofern sich aus der Abbildungslegende nichts anderes ergibt. Sofern das betreffende Material nicht unter der genannten Creative Commons Lizenz steht und die betreffende Handlung nicht nach gesetzlichen Vorschriften erlaubt ist, ist für die oben aufgeführten Weiterverwendungen des Materials die Einwilligung des jeweiligen Rechteinhabers einzuholen. Weitere Details zur Lizenz entnehmen Sie bitte der Lizenzinformation auf http://creativecommons.org/licenses/by/4.0/deed.de.
License URL: https://creativecommons.org/licenses/by/4.0/

issue-copyright-statement: © Springer-Verlag GmbH Deutschland, ein Teil von Springer Nature 2020

==============================
Today, recombinant antibodies can replace animal-derived primary antibodies in almost all applications. Due to their monoclonal origin and always known sequence, they offer optimal reproducibility. In contrast, almost all secondary antibodies are still made from animal sera. Multiclonal antibodies made by animal-free recombinant methods here offer a higher quality replacement for serum-derived secondary antibodies.

■ Der Bericht der EU (ECVAM) zu Ersatzmethoden für Tierversuche [1, 2] zeigt, dass rekombinante Antikörper, welche ohne Immunisierung von Tieren erzeugt wurden, mittlerweile in allen relevanten Anwendungsgebieten der Forschung und Diagnostik angewendet werden und sogar deutliche Vorteile gegenüber tierbasierten Reagenzien bieten. In zahlreichen Veröffentlichungen wurde der Wechsel zu besser definierten Reagenzien bei Antikörperversuchen gefordert [3]. Nur rekombinante Antikörper bieten beides: tierversuchsfreie Herstellung und eine unbegrenzte Reproduzierbarkeit der Ergebnisse, da sie durch ihre Sequenz molekular stets exakt definiert und so unlimitiert nachproduzierbar sind. Tierseren, die derzeit noch den größten Anteil der Forschungsantikörper stellen, sind dagegen irgendwann aufgebraucht. Auch kann ihre stets undefinierte Zusammensetzung aus vielen verschiedenen Antikörpern zu falsch-positiven Ergebnissen führen [4]. Sogar monoklonale Antikörper aus Hybridomen haben oft mehrere unbekannte Spezifitäten, da etwa ein Drittel der Hybridomklone natürlicherweise mehr als eine leichte IgG-Kette produziert [5]. Zwar können die „korrekten" IgG-Moleküle aus Hybridomen identifiziert werden, dies ist aber aufwendig und bei Forschungsreagenzien nicht üblich. Einfacher ist heute die direkte Herstellung neuer Antikörper aus universellen Genbibliotheken mithilfe von Phagen-Display [6]. Damit gelang z. B. in weniger als vier Wochen ohne Tierversuche die Herstellung protektiver menschlicher Antikörper gegen das Coronavirus SARS-CoV-2 [1, 7].

## Multiklonale Antikörper kombinieren die Vorteile polyklonaler, monoklonaler und rekombinanter Antikörper

Während rekombinante, tierversuchsfreie Primärantikörper schon von vielen Anbietern verfügbar sind [2], war es bisher nicht gelungen, so auch Zweitantikörper mit den „polyklonalen" Eigenschaften von Tierseren herzustellen. Die vorteilhaften Eigenschaften der Antikörpergemische in Tierseren können aber unter Vermeidung von deren Nachteilen mit den Vorteilen rekombinanter Antikörper kombiniert werden. Dazu wird eine komplett definierte Mischung von rekombinanten Klonen hergestellt: Diese neue Klasse von Antikörpern wird „multiklonale Antikörper" genannt (**Abb. 1A**). Jeder Einzelantikörper des multiklonalen Gemisches wird dabei tier-

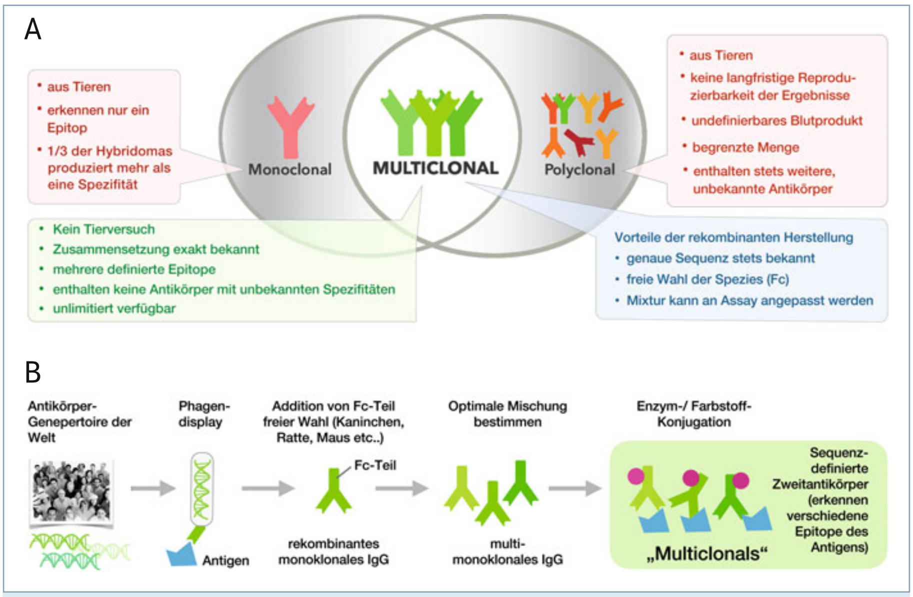

▲ **Abb. 1:** Eigenschaften und Herstellung multiklonaler Antikörper. **A**, Multiklonale Antikörper vereinen die Vorteile der drei bisherigen Antikörperkategorien (polyklonale, monoklonale und rekombinante), vermeiden aber dabei deren jeweilige Nachteile. **B**, Herstellung multiklonaler Antikörper. Aus universellen Genbibliotheken, welche die genetische Gesamtheit der Antikörpersequenzen aus mehr als 100 Spendern enthalten, werden zunächst per Phagen-Display Antikörper gegen verschiedene Epitope des Zielmoleküls hergestellt. Diese werden dann einzeln auf essenzielle Eigenschaften (hohe Affinität, keine Kreuzreaktivität, hohe Stabilität, Funktionalität in bestimmten Assays) getestet. Genau definierte Gemische solcher monoklonaler, sequenzdefinierter Antikörper werden dann gemischt und mit einem Nachweismarker wie Meerrettichperoxidase (*horseradish peroxidase*, HRP) oder Fluoreszenzfarbstoffen konjugiert. Der so entstandene Sekundärantikörper ist im Gegensatz zu den bisher eingesetzten polyklonalen Seren unlimitiert reproduzierbar und enthält keinerlei unbekannte IgGs. Der gesamte Prozess ist zudem komplett tierversuchsfrei.

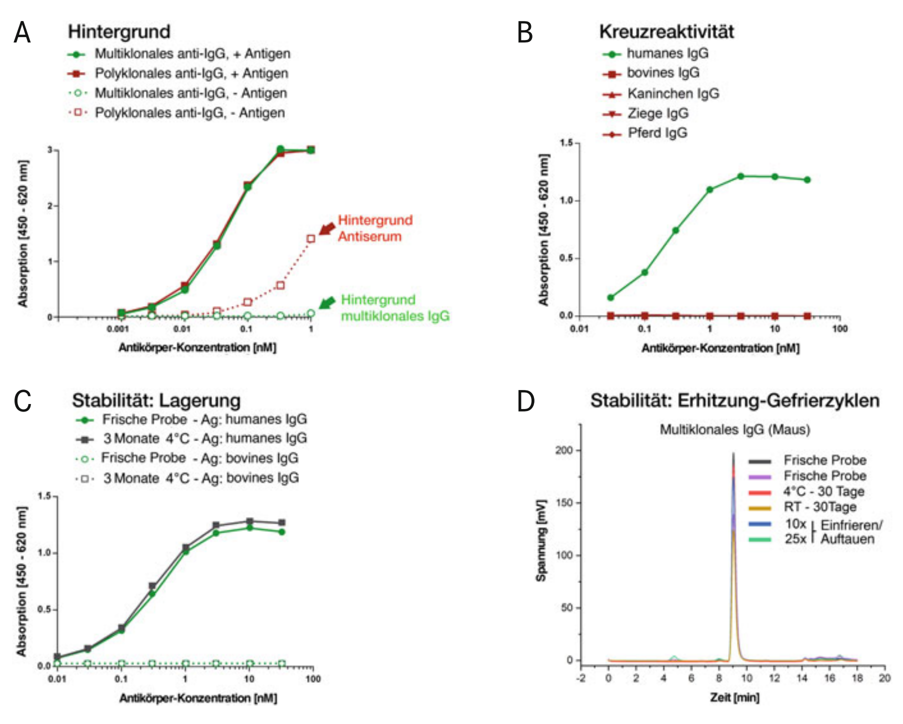

▲ **Abb. 2:** Biochemische Eigenschaften multiklonaler Antikörper. **A**, Bei gleicher Signalstärke erzeugen multiklonale Antikörper ein geringeres Hintergrundsignal als Tierseren (Assay: *Capture*-ELISA). **B**, Spezies-Kreuzreaktivität und **C**, Erhalt der Reaktivität nach Lagerung eines Maus-anti-human-IgG multiklonalen Antikörpers (ELISA). **D**, Die Lagerung für einen Monat bei Raumtemperatur (RT), Erhitzung oder Einfrier-Auftau-Zyklen beeinträchtigen nicht die Qualität eines multiklonalen Antikörpers gegen humanes IgG (Assay: Größenausschluss-Chromatographie).

versuchsfrei durch Phagen-Display aus universellen Genbibliotheken gewonnen, sequenziert und individuell charakterisiert (**Abb. 1B**). Dadurch lassen sich Gemische mit exakt definierten Spezifitätsprofilen zusammenstellen.

Der Vorteil der multiklonalen Antikörper [8] liegt zum einen in der vollständigen Vermeidung von Nebenreaktionen durch die in polyklonalen Seren stets vorhandenen Antikörper unbekannter Spezifitäten, welche zu unerwünschter Hintergrundreaktivität führen können (**Abb. 2A**). Zum anderen kann jeder Antikörper im multiklonalen Gemisch individuell zunächst auf zahlreiche andere relevante Parameter optimiert werden, z. B. auf Kreuzreaktivität mit homologen Proteinen (**Abb. 2B**) oder Stabilität (**Abb. 2C**), um daraus multiklonale Antikörper mit exakt definierter Spezifität zu erzeugen, die nicht aggregieren und lange haltbar sind (**Abb. 2D**).

Die Analyse der EU (ECVAM) zeigt, dass tierbasierte Seren in allen typischen Anwendungen durch tierversuchsfreie und unlimitiert verfügbare rekombinante monoklonale oder multiklonale Antikörper ersetzt werden können. In der Forschung wird die vermehrte Verwendung rekombinanter Antikörper die Reproduzierbarkeit von Versuchen verbessern – mit dem sehr wünschenswerten Nebeneffekt der kompletten Einsparung der heute noch notwendigen Tierversuche zur Antikörperherstellung. In der Diagnostik hängen von der Reproduzierbarkeit der Assays Behandlungsentscheidungen ab – hier sollte sich die Verwendung von Zweitantikörpern mit stets identischem Spezifitätsprofil, die zudem unbegrenzt verfügbar sind, schnell durchsetzen.


Funding

Open Access funding provided durch das Bundesministerium für Wirtschaft und Energie und den Europäischen Sozialfonds im EXIST-Forschungstransfer-Projekt „Abcalis“.

Danksagung

Abcalis wird im Rahmen des EXIST-Forschungstransfers durch das Bundesministerium für Wirtschaft und Energie und den Europäischen Sozialfonds gefördert.


Literatur

[1] Gray A Bradbury A Dübel S Reproducibility: bypass animals for antibody production Nature 2020 581 262 10.1038/d41586-020-01474-7 32415238
[2] Viegas Barroso JF, Halder M, Whelan M (2020) EURL ECVAM Recommendation on Non-Animal-Derived Antibodies. EUR 30185 EN, Publications Office of the European Union, Luxemburg, ISBN 978-92-76-18347-1
[3] Bradbury A Plückthun A Reproducibility: standardize antibodies used in research Nature 2015 518 27 29 10.1038/518027a 25652980
[4] Russo G Theisen U Fahr W Sequence defined antibodies improve the detection of cadherin 2 (N-cadherin) during zebrafish development N Biotechnol 2018 45 98 112 10.1016/j.nbt.2017.12.008 29289749
[5] Bradbury ARM Trinklein ND Thie H When monoclonal antibodies are not monospecific: hybridomas frequently express additional functional variable regions MAbs 2018 10 539 546 10.1080/19420862.2018.1445456 29485921 PMC5973764
[6] Frenzel A Kügler J Helmsing S Designing human antibodies by phage display Transfus Med Hemother 2017 44 312 318 10.1159/000479633 29070976 PMC5649246
[7] Dübel S Hust M Schirrmann T Rekombinante, vollständig humane Antikörper zur Behandlung akuter COVID-19 BlOspektrum 2020 4 444 10.1007/s12268-020-1404-4 PMC7318721 32834543
[8] Abcalis, Abcalis® Multiclonals are a revolutionary new class of secondary antibodies, https://abcalis.com/multiclonals
